# Evaluation of Anxiety, Depression, and Work-Related Strain Inventory of Code Blue Teams in Turkey During the COVID-19 Pandemic: A Cross-Sectional Survey Study

**DOI:** 10.5152/TJAR.2021.21423

**Published:** 2022-08-01

**Authors:** Levent Özdemir, Handan Birbiçer, Nurcan Doruk, Aslınur Sagün

**Affiliations:** 1 Department of Anaesthesiology and Intensive Care, Mersin University Faculty of Medicine, Mersin, Turkey

**Keywords:** Anxiety, code blue, coronavirus disease, depression, mental health

## Abstract

**Objective:**

This study aimed to evaluate the anxiety, depression, and work-related strain inventory with a cross-sectional electronic questionnaire in code blue teams during the coronavirus disease-2019 pandemic in Turkey.

**Methods:**

A web-based electronic questionnaire was sent to healthcare workers registered in the database of the Turkish Society of Anaesthesiology and Reanimation and the Turkish Resuscitation Council who are in the code blue teams of the hospital where they work. An electronic questionnaire including the hospital anxiety-depression scale and the work-related strain inventory was sent to healthcare professionals. A total of 259 participants who answered the questionnaire were included in the study.

**Results:**

It was determined that 41.3% (n = 107) of all participants were at risk in terms of anxiety and 64.1% (n = 166) were at risk in terms of depression by taking above the threshold value. The mean work-related strain inventory score of the participants was found to be 41.19 ± 6.31. The mean work-related strain inventory values of the participants who received above-threshold values from both the anxiety and depression subscales were also found to be statistically significantly higher than the participants who received below-threshold values (*P*  < .001).

**Conclusion:**

It was determined that approximately half of the code blue teams were at risk for anxiety and two-thirds of them for depression.

**Main Points**

- It is important to understand the effects of outbreak periods on the psychological state of healthcare workers, in order to create the necessary precautions and policies.
- During the pandemic, there are very limited studies on psychological diseases that may occur due to fear of contagion in code blue teams, especially due to their applications such as cardiopulmonary resuscitation, including airway management.
- It was determined that approximately half of the code blue teams in Turkey were at risk for anxiety and two-thirds for depression.
- The level of work-related strain was found to be higher in healthcare workers who were at risk for both anxiety and depression.

## Introduction

The novel coronavirus disease (COVID-19), which occurred in the Wuhan region of China, spread rapidly all over the world by being transmitted through droplets and caused acute severe respiratory distress.^1^ Healthcare workers (HCWs) continue to fight on the frontline in the COVID-19 outbreak. Healthcare workers, who care for COVID-19 patients, are at a higher risk for transmission.^2^

The European Resuscitation Council has personal safety recommendations for medical emergency teams (called code blue team [CBT] in Turkey) responding to patients who develop cardiopulmonary arrest while being followed up with a COVID-19 diagnosis.^3^ However, despite all safety precautions, there is a high risk of contamination to HCWs due to the high aerosol generation and the urgency of the clinical situation during the cardiopulmonary resuscitation (CPR) process.^3^ Understanding the psychological impact and prevalence of the COVID-19 outbreak among HCWs is crucial in guiding policies and interventions to maintain their psychological well-being.^4^

Although there are local differences in the CBT in Turkey, the healthcare professionals who make up the team are often anaesthesiologists, intensive care specialists, residents, and nurse anaesthetists. Anxiety, depression, and work-related strain levels can also show different characteristics in CBT formed by healthcare professionals with different education levels and experience.^5^ In this study, the hospital anxiety and depression scale (HADS) and work-related strain inventory (WRSI) were used to evaluate these differences. Since the pandemic still continues, there are very limited studies in the literature on the subject, and no study evaluating the mental status of the CBT has been found.

In this study, it was aimed to evaluate the anxiety, depression, and work-related strain in CBT that intervenes in patients with a diagnosis of COVID-19 with a cross-sectional survey study.

## Methods

Ethics committee approval from Mersin University Clinical Research Ethics Committee (dated 29 April 2020 and numbered 2020/315, Mersin, Turkey) and written permission from the Ministry of Health were obtained for this survey study. In this web-based and cross-sectional survey study, HADS and WRSI scales were applied to HCWs who were members of the CBT in Turkey. This study has been reported according to the principles in the Strengthening the Reporting of Observational Studies in Epidemiology guide.

The members of the CBTs from the healthcare professionals registered in the database of the Turkish Society of Anaesthesiology and Reanimation and Turkish Resuscitation Council were asked to participate in the survey. Participation in the survey was based on voluntariness and an informative letter about the survey was presented in the beginning part of the questionnaire. It was stated that doctors (faculty members, intensive care specialists, residents, and general practitioners) and other HCWs (nurses, nurse anaesthetists, surgical technicians, and emergency medicine technicians) who are members of the CBT in Turkey will be included in the survey. In the information about the questionnaire, it was stated that those with any known psychiatric disease or those using any psychiatric medication should not participate in the questionnaire. A total of 259 participants who answered the questionnaire were included in the study.

The questionnaire form was applied in Turkish language. A questionnaire containing 41 questions was sent to the participants via e-mail. It takes 10 minutes to answer the survey. The survey consisted of demographic characteristics, HADS, and WRSI. The validity and reliability studies in Turkey for both scales (HADS and WRSI) are available in Turkish. All questions were asked as mandatory questions to answer. The questionnaire is web-based, and the responses are also web-based and e-mail address is protected (Appendix 1).

The first 9 questions were asked to collect demographic characteristics. Data on age, gender, job/title, institution where they work, whether they were on the night shift, smoking, marital status, satisfaction with current job, and how they feel when responding to COVID-19 patients were recorded.

Hospital anxiety and depression scale was developed by Zigmond et al^6^ to determine the risk in terms of anxiety and depression in patients and included a total of 14 questions (questions 10-23 in the questionnaire form). It is used to identify those at risk for anxiety or depression in a short time. Of a total of 14 questions, 7 measure anxiety (even-numbered questions starting from the 10th question in the questionnaire form, including the 22nd question) and 7 measure depression (odd-numbered questions starting with the 11th question in the questionnaire and including the 23rd question). Each item is scored on a 4-point Likert-type scale between 0 and 3 points. The lowest score that patients can get from both subscales is 0, and the highest score is 21. In the reliability study of the original article, the Cronbach’s alpha was found to be 0.852 for the anxiety subscale (HAD-A) and 0.778 for the depression subscale (HAD-D). As a result of the receiver operating characteristic (ROC) curve analysis, the cut-off points (threshold score) in the original study of HAD-A were determined as 10 points and 7 points for HAD-D. In the internal consistency analysis of our study, Cronbach's alpha values for HAD-A and HAD-D were found to be 0.719 and 0.681, respectively.

Work-related strain inventory was developed by Revicki et al^7^ in 1991. It is a 4-point Likert-type self-assessment scale with 18 items developed to determine work-related strain in healthcare professionals (questions 24-41 in the questionnaire). Scoring is calculated between 4 and 1 points for each question. Some items are scored in reverse from 1 to 4 (question numbers 25, 27, 31, 32, 34, and 38 in the questionnaire form). The lowest possible score is 18 and the highest score is 72. The scale does not have a cut-off value, and the level of strain due to work changes in direct proportion to the score was obtained from the scale. In the internal consistency analysis of our study, Cronbach's alpha value for WRSI was found to be 0.709.

### Statistical Analysis

Data were analyzed using the Statistical Package for the Social Sciences Version 20.0 (IBM Corp., Armonk, NY, USA). Descriptive statistics for normally distributed data was expressed as mean and standard deviation and for non-normally distributed data, median and interquartile range was employed. Categorical data were expressed as total count and percentages. Shapiro–Wilk test was used for normality. Analytical statistics for normally distributed variables was performed by *t*-test and for non-normally distributed variables, Mann–Whitney test was applied. Categorical variables were analyzed using *χ* ^2^ test. Cronbach's alpha was used for the internal reliability analysis of our survey study. Statistical significance was considered as *P* <.05.

## Results

A total of 259 participants from the CBT who completed the questionnaire were included in the study. The demographic characteristics of the participants are presented in Table 1.

Hospital anxiety and depression scale was evaluated separately for anxiety and depression. It was determined that 41.3% (n = 107) of all participants were in the risk group in terms of anxiety and 64.1% (n = 166) were in the risk group in terms of depression. The WRSI does not have a cut-off value. For similar occupational groups, it offers the opportunity to compare the score determined when applied in different periods. The mean WRSI value of all participants was found to be 41.19 ± 6.31. The results of HAD-A and HAD-D subscales are summarised in Table 2.

For the HAD-A subscale, there was no difference in the mean age of the participants. The number of females who received above the threshold was found to be significantly higher (48.5% vs 28.2%, *P*  = .002). No difference was determined in terms of comparison between profession groups, and the highest rate above the threshold was observed in nurses with 51.6% and residents with 46.6%. When the types of institutions the participants work for are compared, the anxiety rates of those working in university hospitals (47.6%), training and research hospitals (41.6%), and state hospitals (40.0%) were statistically higher than those working in private hospitals (26.3%) and private universities (25.0%). There was no significant difference between the participants with and without night shifts (40.0% vs 41.6%, *P*  = .834). There was no effect of smoking on the HAD-A ratio (41.9% vs 39.3%, *P*  = .721). No significant difference was found according to the marital status of the participants (39.3% for single vs 42.5% for married, *P*  = .845). The HAD-A ratio was found to be statistically significantly higher in participants who were dissatisfied with their current job than those who were satisfied (52.8% vs 30.8%, *P*  < .001).

For the HAD-D subscale, there was no difference in the mean age of the participants. The number of females who got above the threshold was found to be significantly higher (71.2% vs 51.0%; *P*  = .001). No difference was showed in terms of comparison between profession groups, and the highest rate above the threshold was presented in residents with 76.6% and specialist physicians with 60.4%. When the types of institutions the participants work for are compared, the depression rates of those working in university hospitals (67.9%), training and research hospitals (75.0%), state hospitals (62.2%), and private hospitals (52.6%) were statistically higher than those working in private universities (33.3%, *P*  < .001). There was no significant difference between the participants with and without night shifts (60.0% vs 65.0%; *P*  = .834). The HAD-D threshold rate of smokers (52.4%) was significantly lower than non-smokers (67.6%) (*P*  = .03). No significant difference was found according to the marital status of the participants (56.3% for single vs 67.9% for married; *P*  = .07). The HAD-D percentage was found to be statistically significantly higher in those who were dissatisfied with their current job than those who were satisfied (72.3% vs 56.6%; *P*  = .008). When responding to COVID-19 patients, while 80.1% of all participants who scored above the HAD-D threshold felt anxiety, the rate of answering “I do not feel any difference due to protective equipment” was 13.2% of all participants who scored above the HAD-D threshold (*P*  < .001).

The mean WRSI value of all participants was found to be 41.19 ± 6.31 (Table 3). The results of the anxiety–depression subscales were also compared with the WRSI values. The mean WRSI scores of those who scored above the threshold for the anxiety or depression subscales were found to be statistically significantly higher than those for those who were below the threshold (Table 3).

## Discussion

In this study, we evaluated the mental status of healthcare professionals during the COVID-19 outbreak. In our study, approximately half of the healthcare professionals were found to be at risk for anxiety and two-thirds for depression. There are few studies on the mental state of HCWs during the COVID-19 pandemic.^8-10^ Our study is the first to evaluate the level of anxiety, depression, and WRSI in CBT during the COVID-19 outbreak through a cross-sectional questionnaire.

In order to better understand the effects of the pandemic on HCWs’ mental health, it is necessary to review the studies that were conducted when there was no outbreak. In a study by Schmidt et al^11^ when there was no outbreak period, it was found that 31.3% of the nurses showed anxiety symptoms and 24.2% depression symptoms in the HADS score when they applied to the nurses working in surgical wards. A study published by Bentley et al^12^ showed that 6.8% of emergency medical technicians experienced depression and 6.0% for anxiety. In a study conducted by Caplan et al^13^ consulting physicians and general practitioners using HADS scoring, anxiety was found with a rate of 23% in consultant physicians and 30% in general practitioners, while the rate of depression was found to be 19% and 27%, respectively. In another large cross-sectional study conducted among clinicians (n = 2641), it was found that 25.6% showed anxiety symptoms and 28.1% depression symptoms.^14^ Compared with the periods when there was no pandemic, it is seen that the rates of psychological problems documented among HCWs in our study were higher, despite differences such as socioeconomic and ethnic origin. Although our study population consists of healthcare professionals, the fact that it consists of healthcare professionals performing CPR in CBTs may make the situation a little different. It suggests that both the patient's emergency response conditions due to cardiac arrest and airway interventions may increase the risk of transmission. Therefore, fear of the possibility of contamination in CBTs may be more likely to lead to a psychiatric disorder.

Although there is no cut-off value for WRSI, it is accepted that the work-related stress increases as the determined value increases. In our study, the mean WRSI of the participants was found to be 41.19 ± 6.31. In a study conducted among nurses, it was found that the WRSI was reported to have an average score of 38.85 ± 5.76 points.^15^ Factors such as quarantine periods spent away from family, fear of contagion, and night shifts may have been effective in the higher WRSI average as we determined. In our study, it was also observed that those at risk for anxiety or depression had higher work-related tension scores than those who did not.

There are also studies on mental health in epidemics such as Severe Acute Respiratory Syndrome (SARS) and Middle East Respiratory Syndrome (MERS), which have a higher mortality rate than COVID-19 but have no global impact. During the SARS outbreak in 2003, 18%-57% of HCWs were reported to experience severe emotional problems and psychiatric symptoms during and after the epidemic.^16,17^ Tam et al^16^ investigating the mental states of HCWs at the frontline of the SARS outbreak reported the prevalence of stress and psychological problems as 68% and 57%, respectively. Dysphoria and high stress were observed among HCWs during the MERS epidemic in 2015.^18^ Um et al^19^ found that 26.6% of physicians in MERS showed symptoms of depression. Although the results of a study conducted in Singapore suggest that HCW are less mentally affected in the COVID-19 pandemic (14.5% of the participants screened positive for anxiety, 8.9% for depression, and 7.7% for clinical concern of post-traumatic stress disorder)^20^ than previous outbreaks (in 2003, another study conducted in Singapore documented that 20% of participants developed post-traumatic stress disorder after the SARS outbreak),^21^ the authors state that the reason for this may stem from Singapore's mental preparedness and experience resulting from previous outbreaks.^20^ As a result, although there are some differences, the findings of our study support that the probability of mental disorders in HCWs increase, as in previous studies conducted during outbreaks.

According to the type of institution, we obtained lower rates in terms of both anxiety and depression risk in private institutions. When other studies conducted on mental health during the COVID-19 pandemic were reviewed, it was seen that no comparison was made according to the type of institution. The results we obtained are surprising when the literature published in the pre-pandemic period is evaluated. In a study conducted among nurses in 2011, both anxiety and depression rates were found to be higher in nurses in private institutions than in other institution workers.^11^ Similarly, in another study conducted on emergency HCWs in 2013, anxiety and depression rates were found to be higher in private institutions.^12^ This difference may have been caused by the fact that some private hospitals did not accept patients with diagnosed COVID-19 due to the "clean hospital" practice during the pandemic period in Turkey. Also, perhaps, the relatively high salary in private institutions could explain the lower risk of anxiety and depression.

Identifying people with risk factors for anxiety and depression may be important to protect the mental health of healthcare professionals during the pandemic. In a published study, 4 independent variables were found to be associated with anxiety risk among HCW.^22^ These are female gender, living in rural areas, contact with COVID-19 patients in hospitals, and presence of organic disease.^22^ In depression models, being female and the presence of organic disease were identified as independent factors.^22^ In another study, it was found that female gender is more riskier in terms of both anxiety and post-traumatic stress disorder (PTSD).^23^ The data in our study showed that the risk of anxiety and depression is higher in female gender and people who feel stress from contact with COVID-19 patients, and it seems to be in line with the literature.

As another factor that can threaten mental health, there are studies showing that the risk of experiencing anxiety and depressive symptoms is higher in physicians who have 2 or more night shifts per week.^14,24^ In our study, an adverse effect of night shift on mental health was not shown. However, we believe that this result is due to the implementation of flexible working hours in Turkey during the pandemic. In addition, in the data obtained from our study, anxiety and depression symptoms were found with a lower rate in smokers. There is evidence that smokers are less likely to experience anxiety and depressive symptoms, and there are studies showing that nicotine intake can effectively reduce anxiety episodes in particular.^25^

There are some limitations of our study. As with all survey studies, it can be biased because the participants self-report themselves. The socioeconomic, cultural, ethnic differences, and public organisation problems of healthcare professionals included in a study in a geographic region (usually within the borders of a country) may not be similar to healthcare professionals in other regions. In addition, our study population was specific so the results could not be generalised to other healthcare professionals. It was possible for the participants to answer more than once using the survey link. In our study, the fact that the number of female participants is more than male participants may cause bias in the evaluations made in terms of gender. The results of our cross-sectional survey study cannot be attributed solely to the effects of the pandemic. Due to the nature of the cross-sectional study, it was not possible to reveal the cause–effect relationship.

In conclusion, it was determined that approximately half of the CBT that intervened in COVID-19 patients were at risk for anxiety and two-thirds of them for depression. Additionally, the subjects who were at risk for depression or anxiety had higher levels of work-related strain than those not at risk. In our study, it was also determined that female gender, contact with a patient diagnosed or suspected of COVID-19, and being dissatisfied with their current job were risk factors for anxiety, depression, and work-related strain.

## Figures and Tables

**Table 1.** Demographic Characteristics of the Participants

**Features**	**n (%)**
*Age (year),* mean ± SD	37.95 ± 8.80
*Gender* Female Male	167 (64.5) 92 (35.5)
*Profession* Doctors Faculty member Specialist Resident General practitioner Non-physician healthcare workers Nurse Nurse anaesthetits Emergency medical technician Surgical technician	46 (17.8) 96 (37.1) 60 (23.2) 5 (1.9) 31 (12.0) 15 (5.8) 4 (1.5) 2 (0.8)
*Institution (hospitals)* University Private university Training and research State hospital Private hospital	128 (49.4) 12 (4.6) 36 (13.9) 45 (17.4) 38 (14.7)
*Night shift* Yes No	209 (80.7) 50 (19.3)
*Smoking status* Yes No	61 (23.6) 198 (76.4)
*Marital status* Married Single Divorced/widow	162 (62.5) 94 (36.3) 3 (1.2)
*Are you satisfied with your current job?* Satisfied Dissatisfied	136 (52.5) 123 (47.5)
*How do you feel when intervening or following up the patient with diagnosed or suspected COVID-19?* I don’t feel different from other patients because I use personal protective equipment I’m worried about getting infected I am experiencing severe tension or anxiety I have not met such a patient yet I’m not worried because I have recovered from the disease	50 (19.3) 149 (57.5) 37 (14.3) 22 (8.5) 1 (0.4)

SD, standard deviation; COVID-19, coronavirus disease-2019.

**Table 2.** Evaluation of Demographic Characteristics According to HAD-A and HAD-D Scale Results

*Features*	*HAD-A* *Below Threshold (≤10 Points)*	*HAD-A* *Above Threshold* *(>10 Points)*	*P*	*HAD-D* *Below Threshold (≤7 Points)*	*HAD-D* *Above Threshold (>7 Points)*	*P*
Age (mean ± SD)	38.59 ± 9.11	37.03 ± 8.29	.15	38.04 ± 9.19	37.89 ± 8.60	.89
*Gender (n)* Female Male	86 66	81 26	.002	48 45	119 47	.001
*Profession (n)* Doctors Faculty member Specialist Resident General practitioner Non-physician healthcare workers Nurse Nurse anaesthetits Emergency medical technician Surgical technician	29 61 32 3 15 9 0 3	17 35 28 2 16 6 2 1	.46	19 38 14 3 11 6 0 2	27 58 46 2 20 9 2 2	.32
*Institution (hospital) (n)* University Private university Training and research State hospital Private hospital	67 9 21 27 28	61 3 15 18 10	.13	41 8 9 17 18	87 4 27 28 20	.04
*Night shift (n)* Yes No	122 30	87 20	.83	73 20	136 30	.50
*Smoking status (n)* Yes No	37 115	24 83	.72	29 64	32 134	.03
*Marrital status (n)* Married Single Divorced/widow	93 57 2	69 37 1	.84	52 41 0	110 53 3	.07
*Are you satisfied with your current job? (n)* Satisfied Dissatisfied	94 58	42 65	<.001	59 34	77 89	.008
*How do you feel when intervening or following up the patient with diagnosed or suspected COVID-19? (*n*)* I don’t feel different from other patients because I use personal protective equipment I’m worried about getting infected I am experiencing severe tension or anxiety I have not met such a patient yet I’m not worried because I have recovered from the disease	40 89 8 14 1	10 60 29 8 0	<.001	28 49 4 11 1	22 100 33 11 0	<.001

HAD-A, Hospital Anxiety Depression Scale-Anxiety subscale; HAD-D, Hospital Anxiety Depression Scale-Depression subscale; SD, standard deviation; COVID-19, coronavirus disease-2019.

**Table 3.** Comparison of WRSI Results with Demographic Characteristics and Hospital Anxiety–Depression Scale

*Features*	*WRSI,* mean ± SD	*P*
*All participants*	41.19 ± 6.31	*-*
*Gender* Female Male	41.11 ± 6.1441.35 ± 6.65	.77
*Night shift* Yes No	41.63 ± 6.2339.36 ± 6.38	.02
*Smoking status* Yes No	39.25 ± 6.3041.79 ± 6.21	.006
*Marrital status* Married Single Divorced/widow	41.12 ± 6.8141.35 ± 5.4741.82 ± 6.39	.76
*Are you satisfied with your current job?* Satisfied Dissatisfied	38.93 ± 5.5443.69 ± 6.20	<.001
*How do you feel when intervening or following up the patient with diagnosed or suspected COVID-19?* I don’t feel different from other patients because I use personal protective equipment I’m worried about getting infected I am experiencing severe tension or anxiety I have not met such a patient yet I’m not worried because I have recovered from the disease	37.62 ± 6.0242.02 ± 5.8544.46 ± 6.0438.59 ± 6.1033.00	<.001
*HAD-A* Above threshold Below threshold	44.65 ± 5.8638.76 ± 5.44	<.001
*HAD-D* Above threshold Below threshold	43.20 ± 5.6737.60 ± 5.87	<.001

HAD-A, Hospital Anxiety Depression Scale-Anxiety subscale; HAD-D, Hospital Anxiety Depression Scale-Depression subscale; WRSI, work-related strain inventory; SD, standard deviation; COVID-19, coronavirus disease-2019.

**Table:** 

*For each proposition sentence below, please tick the one that suits you best.*				
	**Doesn’t suit me at all**	**Partially suitable for me**	**Suited me greatly**	**Totally suitable for me**
24. Work interferes with family life				
25. My initial job expectations are being realized				
26. I am more edgy than I used to be				
27. I am still the contributor I used to be				
28. I occasionally hide in my office in order shut out others				
29. It seems like I cannot get the recognition that I deserve				
30. I feel guilty when I cannot completely understand my patients or clients				
31. Colleagues at work do contribute their fair share				
32. My productivity has increased				
33. My responsibilities are much different than I had anticipated				
34. My professional growth and skills are continuing				
35. My preoccupation with work makes it hard to disengage from the job at home				
36. I often feel that others are out to take advantage of me				
37. Arguments at home with those close to me have increased recently				
38. I rarely daydream at work				
39. I am working harder but getting less done				
40. Support for my contribution at work has been consistently lacking				
41. I often arrive late for wor**k**				

## Appendix 1. Questionnaire form

**1. Age?**

**2. Gender?**

**3. What is your profession?**

Faculty member (Senior physician)

Specialist

Resident

General practitioner

Nurse

Nurse anesthetist

Surgical technician

Emergency medical technician/Paramedic

**4. The institution you work for? **

University hospital

Private university hospital

Training and research hospital

State Hospital

Private hospital

**5. Do you have a night shift?**

No

Yes

**6. Do you smoke? **

No

Yes

**7. Your marital status? **

Married

Single

Divorced

**8. Are you satisfied with your current job? **

I’m satisfied with my current job

I’m dissatisfied with my current job

**9. How do you feel when intervening or following up the patient with diagnosed or suspected COVID-19? **

I don’t feel different from other patients because I use personal protective equipment.

I’m worried about getting infected

I am experiencing severe tension or anxiety

I have not met such a patient yet

I’m not worried because I have recovered from the disease

**10. I feel tense or ‘wound up’:**

Most of the time

A lot of the time

From time to time, occasionally

Not at all

**11. I still enjoy the things I used to enjoy:**

Definitely as much

Not quite so much

Only a little

Hardly at all

**12. I get a sort of frightened feeling as if something awful is about to happen:**

Very definitely and quite badly

Yes, but not too badly

A little, but it doesn’t worry me

Not at all

**13. I can laugh and see the funny side of things:**

As much as I always could

Not quite so much now

Definitely not so much now

Not at all

**14. Worrying thoughts go through my mind:**

A great deal of the time

A lot of the time

From time to time but not too often

Only occasionally

**15. I feel cheerful:**

Not at all

Not often

Sometimes

Most of the time

**16. I can sit at ease and feel relaxed:**

Definitely

Usually

Not often

Not at all

**17. I feel as if I am slowed down:**

Nearly all the time

Very often

Sometimes

Not at all

**18. I get a sort of frightened feeling like**

‘butterflies’ in the stomach:

Not at all

Occasionally

Quite often

Very often

**19. I have lost interest in my appearance:**

Definitely

I don’t take so much care as I should

I may not take quite as much care

I take just as much care as ever

**20. I feel restless as if I have to be on the move:**

Very much indeed

Quite a l o t

Not very much

Not a t all

**21. I look forward with enjoyment to things:**

As much as ever I did

Rather less than I used to

Definitely less than I used t o

Hardly a t a l l

**22. I get sudden feelings of panic:**

Very often indeed

Quite often

Not very often

Not at all

**23. I can enjoy a good book or radio or TV programme:**

Often

Sometimes

Not often

Very seldom

## References

[1] Zhu N Zhang D Wang W et al. A novel coronavirus from patients with pneumonia in China, 2019. N Engl J Med. 2020;382(8):727 7 33. 10.1056/NEJMoa2001017) 31978945 PMC7092803

[2] Cinar P Kubal T Freifeld A et al. Safety at the time of the COVID-19 pandemic: how to keep our oncology patients and healthcare workers safe. J Natl Compr Canc Netw. 2020;18:1 6. 10.6004/jnccn.2020.7572) 32294617

[3] Nolan JP Monsieurs KG Bossaert L et al. European Resuscitation Council COVID-19 guidelines executive summary. Resuscitation. 2020;153:45 55. 10.1016/j.resuscitation.2020.06.001) 32525022 PMC7276132

[4] Spoorthy MS Pratapa SK Mahant S . Mental health problems faced by healthcare workers due to the COVID-19 pandemic-A review. Asian J Psychiatr. 2020;51:102119. 10.1016/j.ajp.2020.102119) PMC7175897 32339895

[5] Li Z Ge J Yang M et al. Vicarious traumatization in the general public, members, and non-members of medical teams aiding in COVID-19 control. Brain Behav Immun. 2020;88:916 9 19. 10.1016/j.bbi.2020.03.007) 32169498 PMC7102670

[6] Zigmond AS Snaith RP . The hospital anxiety and depression scale. Acta Psychiatr Scand. 1983;67(6):361 3 70. 10.1111/j.1600-0447.1983.tb09716.x) 6880820

[7] Revicki DA May HJ Whitley TW . Reliability and validity of the Work-Related Strain Inventory among health professionals. Behav Med. 1991;17(3):111 1 20. 10.1080/08964289.1991.9937554) 1932844

[8] Kang L Li Y Hu S et al. The mental health of medical workers in Wuhan, China dealing with the 2019 novel coronavirus. Lancet Psychiatry. 2020;7(3):e14. 10.1016/S2215-0366(20)30047-X) PMC7129673 32035030

[9] Xiang YT Yang Y Li W et al. Timely mental health care for the 2019 novel coronavirus outbreak is urgently needed. Lancet Psychiatry. 2020;7(3):228 22 9. 10.1016/S2215-0366(20)30046-8) 32032543 PMC7128153

[10] Muller AE Hafstad EV Himmels JPW et al. The mental health impact of the COVID-19 pandemic on healthcare workers, and interventions to help them: A rapid systematic review. Psychiatry Res. 2020;293:113441. 10.1016/j.psychres.2020.113441) PMC7462563 32898840

[11] Schmidt DR Dantas RA Marziale MH . Ansiedade e depressão entre profissionais de enfermagem que atuam em blocos cirúrgicos [Anxiety and depression among nursing professionals who work in surgical units]. Rev Esc Enferm USP. 2011;45(2):487 4 93. 10.1590/s0080-62342011000200026) 21655802

[12] Bentley MA Crawford JM Wilkins JR Fernandez AR Studnek JR . An assessment of depression, anxiety, and stress among nationally certified EMS professionals. Prehosp Emerg Care. 2013;17(3):330 33 8. 10.3109/10903127.2012.761307) 23414106

[13] Caplan RP Stress, anxiety, and depression in hospital consultants, general practitioners, and senior health service managers. BMJ. 1994;309(6964):1261 126 3. 10.1136/bmj.309.6964.1261) 7888846 PMC2541798

[14] Gong Y Han T Chen W et al. Prevalence of anxiety and depressive symptoms and related risk factors among physicians in China: A cross-sectional study. PLOS ONE. 2014;9(7):e103242. 10.1371/journal.pone.0103242) PMC4106870 25050618

[15] Erdoğan C Doğan S Çakmak R et al. Assessment of job satisfaction, work-related strain, and perceived stress in nurses working in different departments in the same hospital: a survey study. Ain-Shams J Anesthesiol. 2020;12(1):34. 10.1186/s42077-020-00084-9)

[16] Tam CW Pang EP Lam LC Chiu HF . Severe acute respiratory syndrome (SARS) in Hong Kong in 2003: stress and psychological impact among frontline healthcare workers. Psychol Med. 2004;34(7):1197 1 204. 10.1017/s0033291704002247) 15697046

[17] Nickell LA Crighton EJ Tracy CS et al. Psychosocial effects of SARS on hospital staff: survey of a large tertiary care institution. CMAJ. 2004;170(5):793 79 8. 10.1503/cmaj.1031077) 14993174 PMC343853

[18] Xiao J Fang M Chen Q He B . SARS, MERS and COVID-19 among healthcare workers: A narrative review. J Infect Public Health. 2020;13(6):843 84 8. 10.1016/j.jiph.2020.05.019) 32493671 PMC7250777

[19] Um DH Kim JS Lee HW Lee SH . Psychological effects on medical doctors from the Middle East respiratory syndrome (MERS) outbreak: A comparison of whether they worked at the MERS occurred hospital or not, and whether they participated in MERS diagnosis and treatment. J Korean Neuropsychiatr Assoc. 2017;56(1):28 34. 10.4306/jknpa.2017.56.1.28)

[20] Tan BYQ Chew NWS Lee GKH et al. Psychological impact of the COVID-19 pandemic on health care workers in Singapore. Ann Intern Med. 2020;173(4):317 320. 10.7326/M20-1083) 32251513 PMC7143149

[21] Chan AO Huak CY . Psychological impact of the 2003 severe acute respiratory syndrome outbreak on health care workers in a medium size regional general hospital in Singapore. Occup Med (Lond). 2004;54(3):190 19 6. 10.1093/occmed/kqh027) 15133143 PMC7107861

[22] Zhang WR Wang K Yin L et al. Mental health and psychosocial problems of medical health workers during the COVID-19 epidemic in China. Psychother Psychosom. 2020;89(4):242 2 50. 10.1159/000507639) 32272480 PMC7206349

[23] Huang JZ Han MF Luo TD Ren AK Zhou XP . Mental health survey of medical staff in a tertiary infectious disease hospital for COVID-19. Zhonghua Lao Dong Wei Sheng Zhi Ye Bing Za Zhi. 2020;38(3):192 19 5. 10.3760/cma.j.cn121094-20200219-00063) 32131151

[24] Bannai A Tamakoshi A . The association between long working hours and health: a systematic review of epidemiological evidence. Scand J Work Environ Health. 2014;40(1):5 18. 10.5271/sjweh.3388) 24100465

[25] Pomerleau OF Pomerleau CS . Behavioural studies in humans: anxiety, stress and smoking. Ciba Found Symp. 1990;152:225 35; discussion 235. 10.1002/9780470513965.ch13) 2209256

